# myomiR-dependent switching of BAF60 variant incorporation into Brg1 chromatin remodeling complexes during embryo myogenesis

**DOI:** 10.1242/dev.108787

**Published:** 2014-09

**Authors:** Katarzyna Goljanek-Whysall, Gi Fay Mok, Abdulmajeed Fahad Alrefaei, Niki Kennerley, Grant N. Wheeler, Andrea Münsterberg

**Affiliations:** School of Biological Sciences, University of East Anglia, Norwich Research Park, Norwich NR4 7TJ, UK

**Keywords:** BAF chromatin remodeling complex, Brg1, miR-1, miR-206, miR-133, Smarcd, Chick embryo, Somite myogenesis

## Abstract

Myogenesis involves the stable commitment of progenitor cells followed by the execution of myogenic differentiation, processes that are coordinated by myogenic regulatory factors, microRNAs and BAF chromatin remodeling complexes. BAF60a, BAF60b and BAF60c are structural subunits of the BAF complex that bind to the core ATPase Brg1 to provide functional specificity. BAF60c is essential for myogenesis; however, the mechanisms regulating the subunit composition of BAF/Brg1 complexes, in particular the incorporation of different BAF60 variants, are not understood. Here we reveal their dynamic expression during embryo myogenesis and uncover the concerted negative regulation of BAF60a and BAF60b by the muscle-specific microRNAs (myomiRs) miR-133 and miR-1/206 during somite differentiation. MicroRNA inhibition in chick embryos leads to increased BAF60a or BAF60b levels, a concomitant switch in BAF/Brg1 subunit composition and delayed myogenesis. The phenotypes are mimicked by sustained BAF60a or BAF60b expression and are rescued by morpholino knockdown of BAF60a or BAF60b. This suggests that myomiRs contribute to select BAF60c for incorporation into the Brg1 complex by specifically targeting the alternative variants BAF60a and BAF60b during embryo myogenesis, and reveals that interactions between tissue-specific non-coding RNAs and chromatin remodeling factors confer robustness to mesodermal lineage determination.

## INTRODUCTION

Myogenesis in vertebrate embryos serves as a paradigm for cell fate commitment. The signals leading to the activation of myogenic regulatory factors (MRFs) *in vivo*, in the myotome of developing somites, are well characterized (Mok and Sweetman, 2011). Following myogenic commitment, a hierarchy of transcription factors controls the myogenic program (Bajard et al., 2006; Buckingham and Rigby, 2014).

An important feature of muscle development and regeneration is post-transcriptional gene regulation by microRNAs (miRNAs), which are short non-coding RNAs that bind to target sites within mRNAs typically located in 3′UTRs (Williams et al., 2009a; Goljanek-Whysall et al., 2012a). miRNAs act through inhibition of translation and promote the degradation of target transcripts (Bartel, 2009; Bethune et al., 2012), suggesting a role for miRNAs in conferring accuracy of developmental timing and in supporting cell fate decisions and tissue identity (Stark et al., 2005; Hornstein and Shomron, 2006; Mann et al., 2010; Ebert and Sharp, 2012).

In skeletal muscle, two highly conserved miRNA families, miR-1/206 and miR-133, play important roles in proliferation, differentiation and cell fate specification; therefore, they have been termed myomiRs (McCarthy, 2008; van Rooij et al., 2008). In vertebrate embryos, miR-206 expression is restricted to skeletal myoblasts in somites, limb buds and head muscles, whereas miR-1 and miR-133 are expressed in developing skeletal muscle and heart (Darnell et al., 2006; Sweetman et al., 2006, 2008). In somites and C2C12 myoblasts, MRFs regulate miR-1, miR-206 and miR-133 expression (Rao et al., 2006; Rosenberg et al., 2006; Sweetman et al., 2008). miRNA-mediated negative regulation of target mRNAs is important for myogenic differentiation of C2C12 myoblasts, and the sustained expression of some miR-1/206 targets results in the activation of non-myogenic programs (Goljanek-Whysall et al., 2012b). In developing embryos, miR-1 and miR-206 have been shown to facilitate myogenic differentiation through negative regulation of the paired-box transcription factor Pax3 in myogenic progenitor cells (Goljanek-Whysall et al., 2011). This interaction is recapitulated during the activation of adult muscle stem cells (Chen et al., 2010; Hirai et al., 2010).

Members of the miR-1/206 family are produced from the same primary transcripts as members of the miR-133 family. In addition, these miRNAs are produced from multiple genomic loci: three in mouse and human and four in chick, which makes genetic approaches in mice challenging. Individual deletion of miR-1-2 or miR-206 does not lead to an overt skeletal muscle phenotype in adult mice (Zhao et al., 2007; Williams et al., 2009b). However, the regenerative capacity of skeletal muscle is compromised and loss of miR-206 attenuates muscle degenerative phenotypes seen in models of amyotrophic lateral sclerosis (ALS) and Duchenne muscular dystrophy (DMD) (Williams et al., 2009b; Liu et al., 2012). Genetic deletion of miR-133a-1 and miR-133a-2 in muscle leads to an adult-onset centronuclear myopathy, which correlates with the dysregulation of dynamin 2 (DNM2). This illustrates the essential role of miR-133a in the maintenance of adult skeletal muscle structure and myofiber identity (Liu et al., 2011). In embryonic stem cells (ESCs), miR-1 and miR-133 promote mesoderm differentiation (Ivey et al., 2008), and transcriptomic analyses in zebrafish have revealed their importance for sarcomeric actin organization (Mishima et al., 2009). The chick embryo allows loss-of-function studies using the targeted microinjection of antagomirs, which are powerful inhibitors of miRNA function (Krutzfeldt et al., 2005; McGlinn et al., 2009). We previously used this approach to uncover a requirement for miR-206 and, to a lesser degree, for miR-1 activity for Pax3 downregulation in the somite myotome, which ensures the timely transition of myogenic progenitor to committed myoblast (Goljanek-Whysall et al., 2011).

Chromatin remodeling determines access to gene regulatory elements by the transcriptional machinery and is thus important for lineage determination, including myogenic specification. The BAF chromatin remodeling complexes are important in neural and skeletal muscle differentiation and consist of 11 core subunits (Kadam and Emerson, 2003; Puri and Mercola, 2012). Combinatorial assembly of alternative BAF subunits together with the ATPase Brg1 leads to diversity, which is proposed to confer functional specificity in both neural and skeletal muscle lineages (de la Serna et al., 2001; Lessard et al., 2007; Wu et al., 2009; Yoo and Crabtree, 2009).

Mammalian cells can express three variants of the BAF60 subunit, which are encoded by different genes: BAF60a (Smarcd1), BAF60b (Smarcd2) and BAF60c (Smarcd3). In mouse and zebrafish BAF60c is expressed in developing heart, somites and neural tube (Lickert et al., 2004; Lamba et al., 2008). BAF60c is essential for cardiac and skeletal myogenesis and promotes the activation of cardiac and skeletal muscle-specific genes, including muscle-specific miRNAs (Lickert et al., 2004; Ochi et al., 2008; Mallappa et al., 2010). During cardiogenesis, BAF60c interacts with the cardiac-specific transcription factor GATA4 (Lickert et al., 2004; Takeuchi and Bruneau, 2009), whereas during skeletal myogenesis BAF60c interacts with MyoD, a key regulator of myogenesis (Forcales et al., 2012). Different BAF60 variants are present in distinct mammalian BAF complexes (Wang et al., 1996); for example, BAF60a but not BAF60c is present in mouse ESCs (Ho et al., 2009). In muscle, incorporation of BAF60a or BAF60b into the BAF complex might inhibit its ability to respond to pro-myogenic signaling (Forcales et al., 2012), and we recently showed that BAF60b activates alternative, non-myogenic differentiation programs in C2C12 cells, including chondrogenesis and osteogenesis (Goljanek-Whysall et al., 2012b). BAF subunit composition produces biological specificity; however, the mechanisms regulating BAF/Brg1 complex assembly during embryonic muscle development are poorly understood.

Here, using complementary *in vitro* and *in vivo* assays, we identified BAF60a and BAF60b as key targets of the myomiRs miR-1/206 and miR-133 during initiation of the myogenic differentiation program in embryogenesis. Injection of antagomirs into somites of developing chick embryos led to increased levels of BAF60a and BAF60b transcript and protein. This in turn affected the incorporation of BAF60 variants into BAF/Brg1 complexes and impaired the timing of myoblast differentiation *in vivo*. Sustained expression of either BAF60a or BAF60b mimicked the phenotype induced by antagomirs. Rescue experiments showed that myogenesis was restored in antagomir-injected somites by morpholino-mediated BAF60a or BAF60b knockdown. We propose that, following myoblast commitment, miRNA-mediated post-transcriptional repression of residual *BAF60a* and *BAF60b* transcripts is a key event by which miR-133 and miR-1/206 stabilize the myogenic differentiation program in the embryo.

## RESULTS

### BAF60 variants are dynamically expressed during somite development

**Fig. 1.**
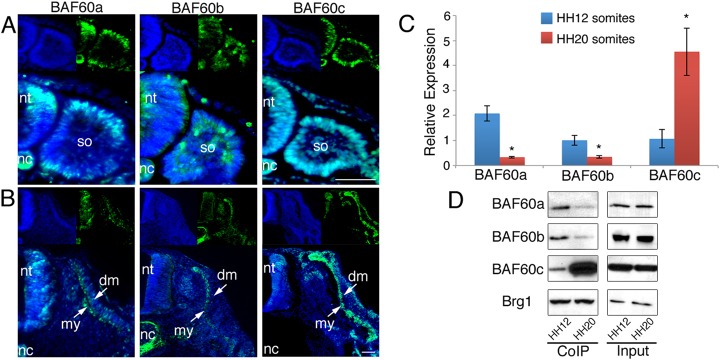
**Expression of BAF60 variants and Brg1/BAF complex composition during somite development.** (A) Immunohistochemistry on somite sections of HH12 chick embryos illustrates the expression of all BAF60 variants (green) in epithelial somites. (B) Immunohistochemistry on somite sections of HH20 embryos shows expression of all BAF60 variants in the myotome. DAPI stain (blue) shows cell nuclei. Scale bars: 50 μm. (C) qPCR of HH12 and HH20 somites shows that relative amounts of *BAF60a* and *BAF60b* transcripts are decreasing, whereas transcripts encoding BAF60c are increasing during development. (D) CoIP using anti-Brg1 antibody and protein isolated from HH12 or HH20 somites. The amount of BAF60a and BAF60b protein bound to Brg1 decreases over time, whereas the amount of BAF60c variant associated with Brg1 increases in differentiating somites. Input samples are shown. dm, dermomyotome; my, myotome; nc, notochord; nt, neural tube; so, somite.

To examine their role in myogenesis, we investigated the expression of BAF60a, BAF60b and BAF60c transcripts and protein *in vivo* using chick embryos from Hamburger–Hamilton (HH; Hamburger and Hamilton, 1992) stage 12 to 20 (Fig. 1A-C; supplementary material Fig. S1A,B). Prior to myotome formation (HH12), BAF60a, BAF60b and BAF60c proteins were detected throughout immature epithelial somites (Fig. 1A); peptide blocking experiments indicate antibody specificity (supplementary material Fig. S1C). Somites undergo complex morphogenesis and the dorsal part forms the epithelial dermomyotome, from which cells enter the myotome in successive waves and initiate myogenic differentiation (Gros et al., 2004). In maturing somites (HH20), BAF60a, BAF60b and BAF60c were all expressed in the myotome (Fig. 1B). Quantitative PCR (qPCR) showed that relative transcript amounts were similar for all three BAF60 variants in epithelial somites isolated from HH12 embryos, whereas in differentiating somites from HH20 embryos *BAF60c* expression was increased and *BAF60a* and *BAF60b* were expressed at low levels (Fig. 1C).

To determine how differential expression of BAF60a, BAF60b and BAF60c variants in developing somites affects BAF/Brg1 complex composition, we performed co-immunoprecipitations (CoIPs) using antibody against the core subunit Brg1 (Simone et al., 2004; Forcales et al., 2012). In epithelial somites at HH12, which consist of unspecified lineage precursors, all three BAF60 subunits bound to Brg1 at comparable levels. In somites of HH20 embryos, where lineage commitment has begun, the amount of BAF60c protein that co-immunoprecipitated with Brg1 was increased, whereas the amounts of BAF60a and BAF60b variants present in the complex were decreased compared with HH12 (Fig. 1D; supplementary material Fig. S1D), indicating a switch in Brg1/BAF60 subunit composition during somite maturation.

### Knockdown of BAF60c or targeted misexpression of BAF60a/BAF60b abrogates myogenesis

**Fig. 2.**
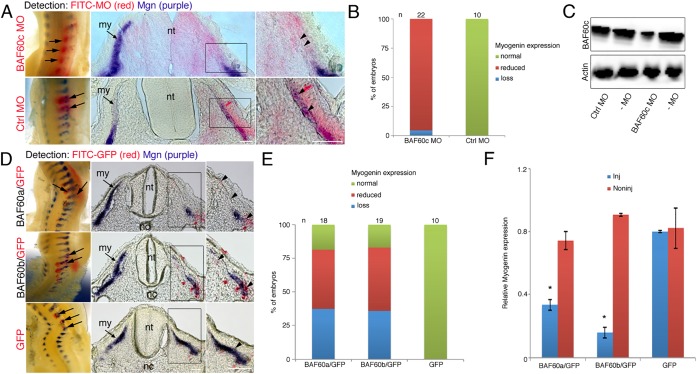
**MO knockdown of BAF60c or misexpression of BAF60a or BAF60b variants inhibits myogenesis.** (A) Electroporation of BAF60c-MO or control-MO into somites on one side, followed by *in situ* hybridization for myogenin (purple) and detection of the FITC-coupled MO (red). Whole-mount views and sections show that BAF60c-MO led to localized myogenin loss (arrows and arrowheads), whereas control-MO had no effect. (B) Percentage of embryos with an effect on myogenin expression after BAF60c-MO injection. (C) Western blot of pooled somites shows reduced BAF60c protein levels in BAF60c-MO electroporated somites, when compared with control-MO or to somites from the non-injected side (–MO). MO electroporations are mosaic and images shown in A give a spatial resolution, whereas the western blot in C averages what occurs in all cells across the somite. (D) Whole-mount double *in situ* hybridization and sections show that misexpression of BAF60a or BAF60b variants in somites leads to localized loss of myogenin expression. Myogenin is in purple (arrows and arrowheads) and *GFP* transcripts, which are expressed from a separate, co-injected plasmid, are in red. The ratio of BAF60 expression plasmid to GFP expression plasmid is 5:1. (E) Myogenin expression phenotypes observed after electroporation. (F) qPCR shows reduced myogenin expression in somites electroporated with BAF60a or BAF60b expression vectors when compared with GFP plasmid controls. Material from multiple embryos was pooled. Error bars indicate s.d.; *P<0.05 (*t*-test). my, myotome; nt, neural tube; nc, notochord. Scale bars: 50 μm.

BAF60c is important for myogenic differentiation of C2C12 myoblasts (Forcales et al., 2012). To assess the requirement of BAF60c for embryo myogenesis we used a knockdown (KD) approach in somites from HH14-15. Somites were analyzed 24 h after electroporation of specific FITC-labeled antisense morpholinos (MOs). Electroporation of BAF60c-MO led to localized loss of myogenin, a skeletal muscle differentiation marker, indicating that myogenic differentiation was inhibited on the injected side (Fig. 2A,B). Expression of myogenin was unaffected in control-MO-injected somites (Fig. 2A,B) or in somites injected with BAF60a-MO or BAF60b-MO (supplementary material Fig. S2A,B). Western blot of pooled somites, which averages the amount of protein present across the tissue, showed reduced BAF60a, BAF60b and BAF60c proteins after injection of BAF60 MOs compared with non-injected somites and control-MO (Fig. 2C; supplementary material Fig. S2C). This suggests that BAF60a and BAF60b variants are dispensable for somite myogenesis and confirms the importance of BAF60c in this process, consistent with findings in mice in which BAF60c was shown to be important for cardiac and skeletal muscle development after siRNA KD (Lickert et al., 2004).

We next asked whether sustained expression of BAF60a and BAF60b adversely affects myogenesis. Epithelial somites were electroporated at HH14-15 with BAF60a or BAF60b expression vectors together with a trace amount of GFP plasmid, or with GFP plasmid alone, and analyzed after 24 h (Fig. 2D). Increased expression of *BAF60a* and *BAF60b* was confirmed by qPCR (supplementary material Fig. S2D,E). GFP electroporation alone had no effect on myogenic differentiation. By contrast, expression of myogenin was completely or partially reduced 24 h after targeted misexpression of BAF60a or BAF60b. Phenotypes of embryos were assessed after whole-mount *in situ* hybridization; selected embryos were processed for cryosections (Fig. 2D,E). Effects on myogenic differentiation were confirmed by qPCR of pooled transfected somites, which had reduced myogenin transcript levels compared with controls (Fig. 2F).

These results show that differential expression of BAF60 variants in embryonic somites is important for myogenic differentiation and indicate that sustained expression of BAF60a and BAF60b interferes with BAF60c function. Thus, we examined factors that might negatively regulate BAF60a and BAF60b in the developing myotome.

### BAF60a and BAF60b expression in developing somites is negatively regulated by myomiRs

**Fig. 3.**
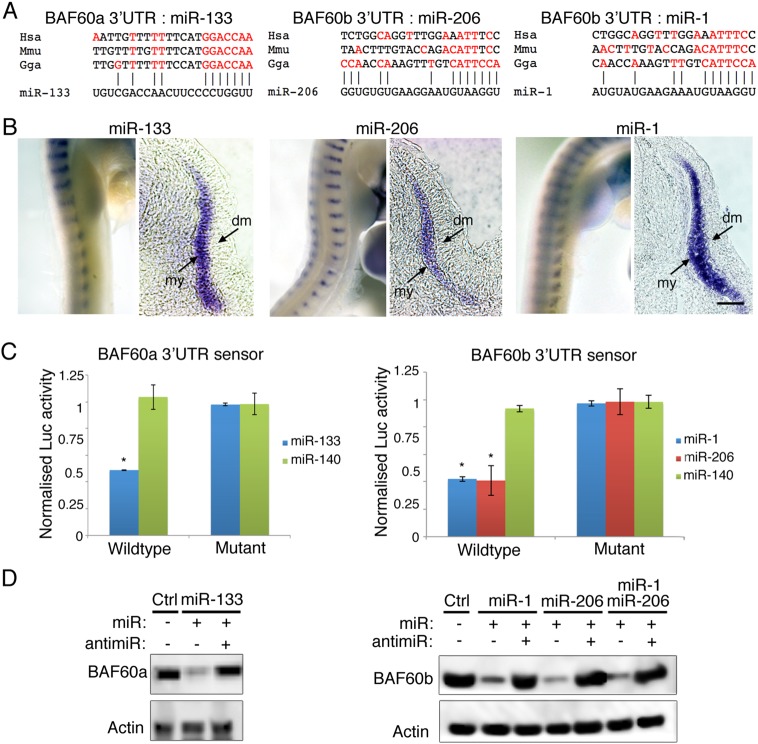
**miR-133 and miR-1/206 regulate the expression of BAF60a and BAF60b variants.** (A) Alignment of putative miR-133, or miR-206 and miR-1 target sites in the 3′UTRs of chick (*Gallus gallus*, Gga), human (*Homo sapiens*, Hsa) and mouse (*Mus musculus*, Mmu) BAF60a and BAF60b genes. Nucleotides complementary to the respective miRNA are in red. The seed sequence of miR-133 is complementary to the predicted target site in the *BAF60a* 3′UTR and this is conserved in all three species. There is little variation outside the seed sequence. The seed sequences of miR-1 and miR-206, which are identical, are complementary to the predicted target site in the chicken *BAF60b* 3′UTR. In human and mouse *BAF60b* 3′UTR, fewer nucleotides are complementary to the miR-1 or miR-206 seed sequence, suggesting a non-canonical binding site where nucleotides outside the seed compensate. The number and position of complementary nucleotides outside the seed sequence vary between species and between miR-1 and miR-206, as these miRNAs differ outside the seed. In human/mouse, *BAF60a* and *BAF60b* are known as *SMARCD1*/*Smarcd1* and *SMARCD2*/*Smarcd2*, respectively. (B) *In situ* hybridization using LNA probes shows myotome-specific expression of miR-133, miR-206 and miR-1 in HH20 embryos. my, myotome; dm, dermomyotome. Scale bar: 50 μm. (C) Luciferase sensors containing 3′UTR sequences of chick *BAF60a* or *BAF60b* were transfected into DF1 cells. Co-transfection of miR-133, miR-1 or miR-206 led to downregulation of luciferase expression compared with controls. Point mutations in the putative target site rendered the sensors non-responsive. Error bars indicate s.d.; *P<0.05 (*t*-test). (D) Endogenous BAF60a and BAF60b proteins are regulated by miR-133 or miR-1/206 in mouse NIH3T3 cells. Transfection with miR-133 led to reduced BAF60a protein levels; co-transfection of miR-133 with antimiR-133 restored BAF60a protein levels to that of mock transfected controls. Transfection with miR-1 or miR-206 or both led to reduced BAF60b protein levels; co-transfection of miRNAs with the relevant antimiR restored BAF60b protein levels to that of mock transfected controls.

Alignments show that a putative miR-133 binding site, with a seed sequence conserved between chick (*Gallus gallus*, Gga), human (*Homo sapiens*, Hsa) and mouse (*Mus musculus*, Mmu), is present in the 3′UTR of the *BAF60a* gene. A putative binding site for miR-1 or miR-206 was found in the 3′UTR of the *BAF60b* gene, but here the seed sequence is less well conserved between the three species (Fig. 3A). *In situ* hybridization shows that miR-1, miR-206 and miR-133 are expressed in the myotome (Fig. 3B; see also Goljanek-Whysall et al., 2011; Sweetman et al., 2008). This correlates with reduced *BAF60a* and *BAF60b* transcripts and with less BAF60a and BAF60b bound to Brg1 in differentiating somites (Fig. 1C,D). To test whether BAF60a and BAF60b are directly targeted by miR-133 and by miR-1 or miR-206 respectively, sensor constructs were generated with 3′UTR fragments containing putative miRNA binding sites downstream of luciferase (Fig. 3C). Co-transfection of a BAF60a sensor with miR-133 led to downregulation of luciferase expression compared with co-transfection with miR-140, an unrelated miRNA not predicted to target the 3′UTR. Similarly, co-transfection of a BAF60b sensor with miR-1 or miR-206 led to downregulation of luciferase expression compared with controls. Point mutations introduced into the putative target sites rendered sensors non-responsive to the respective miRNAs (Fig. 3C). The chicken BAF60a 3′UTR sensor did not respond to miR-1 or miR-206, and the BAF60b 3′UTR sensor did not respond to miR-133 (supplementary material Fig. S3C).

To examine the regulation of endogenous *BAF60a* and *BAF60b* transcripts by miRNAs in a physiological context and in a different species we used mouse NIH3T3 cells, which express all BAF60 variants (supplementary material Fig. S3B), but do not express the myomiRs miR-133, miR-1 or miR-206. Western blots show that transfection with miR-133 led to reduced BAF60a protein compared with control cells, and co-transfection of antimiR-133 restored BAF60a protein levels to that of controls (Fig. 3D). Transfection with miR-1 or miR-206 or both miRNAs led to reduced BAF60b protein compared with control cells, and co-transfection with antimiR-206, which inhibits both miR-1 and miR-206 (Goljanek-Whysall et al., 2011), restored BAF60b protein levels to that of controls (Fig. 3D). The effect of miR-133 or a combination of miR-1 and miR-206 on transcript levels of all BAF60 variants was also examined by qPCR. This confirmed that miR-133 transfection led to reduced *BAF60a* expression but did not affect the levels of the other two variants. Similarly, miR-1/206 transfection resulted in lower levels of endogenous *BAF60b* expression without affecting *BAF60a* or *BAF60c*. Expression of *BAF60c* was unaffected by miR-133 or miR-1/206 (supplementary material Fig. S3B).

### Inhibition of miR-133 or miR-1/miR-206 abrogates myogenesis and alters BAF/Brg1 subunit composition

**Fig. 4.**
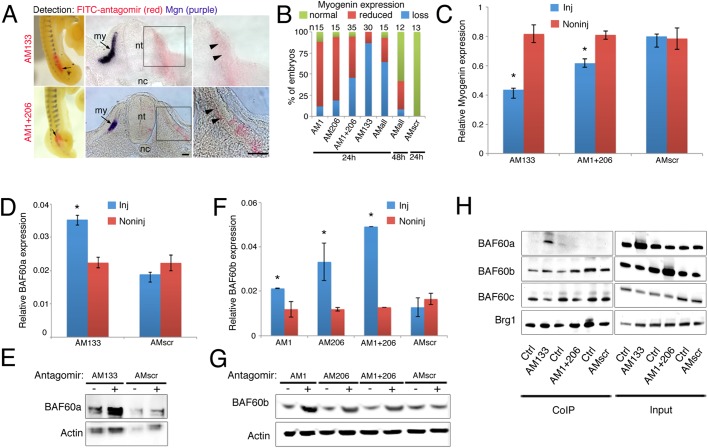
**Inhibition of miRNAs abrogates myogenesis and alters Brg1/BAF subunit composition.** (A) Antagomir (AM) injections followed by *in situ* detection of myogenin transcripts (purple) and FITC-labeled AMs (red) shows loss or reduction of myogenin expression on the injected side (arrows and arrowheads). Whole-mounts and sections are shown. my, myotome; nt, neural tube; nc, notochord. Scale bars: 50 μm. (B) Percentage of embryos that displayed normal, reduced or no myogenin expression following different AM injections. AMall, all three AMs combined; AMscr, scrambled AM. Embryos were incubated for 24 or 48 h as indicated. (C) qPCR detecting relative amounts of myogenin transcripts in somites injected as indicated. (D) qPCR shows increased *BAF60a* expression in somites injected with AM133 compared with non-injected somites. Somites injected with AMscr do not have elevated *BAF60a* levels compared with non-injected somites. (E) BAF60a was detected by western blot in somites injected with AM133 or AMscr; (−) indicates non-injected control side. BAF60a variant was elevated after miR-133 inhibition. (F) qPCR shows increased *BAF60b* expression in somites injected with AM1, AM206, or both, compared with non-injected somites. Somites injected with AMscr do not have elevated *BAF60b* levels compared with non-injected somites. (C,D,F) Error bars indicate s.d.; *P<0.05 (*t*-test). (G) BAF60b detected by western blot in somites injected as indicated; (−) indicates non-injected side. BAF60b variant was elevated after miR-1/miR-206 inhibition. (H) CoIP using anti-Brg1 antibody and protein extracts from somites injected with AM133, versus non-injected somites, shows increased amounts of BAF60a protein complexed with Brg1 in the AM133-injected sample. The amount of BAF60c variant that co-precipitated with Brg1 was reduced in the AM133-injected sample (lanes 1, 2). CoIP using Brg1 antibody and protein extracts from somites injected with AM1 and AM206, versus non-injected control, shows an increased amount of BAF60b protein complexed with Brg1 in the AM1- and AM206-injected sample. The amount of BAF60c variant that co-precipitated with Brg1 was reduced in the AM1- and AM206-injected sample (lanes 3, 4). CoIP using anti-Brg1 antibodies and protein extracts from somites injected with AMscr, or from non-injected somites, show that similar amounts of BAF60 variants were complexed with Brg1 in both samples (lanes 5, 6). Input samples (lanes 7-12) show increased BAF60a and BAF60b after AM133 and AM1/206 injections, respectively.

We next examined the consequences of antagomir-mediated inhibition of miRNA function for both embryo myogenesis and BAF60a and BAF60b expression levels. The function of miR-133 or miR-1/miR-206 was inhibited by injection of specific antagomirs into somites of HH14-15 embryos, which were analyzed after 24 h. Northern blots of pooled somites showed that antagomir-133 inhibited miR-133 expression (supplementary material Fig. S4A). We previously showed that antagomir-1 or antagomir-206 specifically inhibits miR-1 or miR-206, respectively (Goljanek-Whysall et al., 2011). Furthermore, PCR experiments showed that antagomir-1 and antagomir-206 led to loss of miR-1 and miR-206, but had no effect on miR-133, and antagomir-133 specifically affected miR-133 and had no effect on miR-1 or miR-206 (supplementary material Fig. S4B,C). Inhibition of miR-1 or miR-206 at this stage led to partial loss of myogenin expression in the majority of embryos as compared with the contralateral control (Fig. 4A,B). Simultaneous inhibition of both miR-1 and miR-206 led to a significant number of embryos with complete loss of myogenin expression (Fig. 4B, third column). Because of this more prominent phenotype, we used a combination of antagomir-1 and antagomir-206 in all further experiments.

We next analyzed the effects of miR-133 inhibition in maturing somites. Interestingly, antagomir-mediated inhibition of miR-133 in somites led to complete loss of myogenin expression in the majority of embryos (85%). In addition, somite morphology was altered and both dermomyotome and myotome were poorly defined (Fig. 4A,B fourth column). Injection of scrambled antagomir had no effect on embryo myogenesis (Fig. 4B, seventh column; supplementary material Fig. S4D). qPCR analyses of pooled somites injected with specific antagomirs demonstrated significantly reduced myogenin expression compared with non-injected or scrambled-injected control somites (Fig. 4C). Inhibition of all three myomiRs in developing somites led to loss of myogenin expression in the majority of embryos after 24 h (Fig. 4B, fifth column). After 48 h, myogenesis was still impaired in embryos injected with a combination of antagomirs (antagomir-1, -206 and -133) even though effects were less severe (Fig. 4B, sixth column). This indicates a role for myomiRs in controlling entry into the myogenic differentiation program during embryogenesis.

Next we investigated the regulation of BAF60a and BAF60b expression by myomiRs *in vivo*. Specific antagomirs inhibiting miR-133 or miR-1/206 were injected into somites on one side of the embryo at HH14-15. We then examined BAF60a and BAF60b transcript and protein levels. Non-injected somites from the contralateral side and scrambled antagomir-injected somites served as controls. Microinjection of antagomir-133 led to increased BAF60a transcript and protein levels in somites compared with controls (Fig. 4D,E). Microinjection of either antagomir-1 or antagomir-206, or a mixture of both antagomirs, led to increased BAF60b transcript and protein levels compared with controls (Fig. 4F,G).

Finally, we examined whether increased BAF60a and BAF60b protein levels, after antagomir-mediated inhibition of miR-133 or miR-1/206, altered the composition of BAF/Brg1 complexes. To assess whether relative amounts of BAF60 variants were affected, we performed CoIPs using anti-Brg1 antibody. In antagomir-133-injected somites, the amount of BAF60a found in a complex with Brg1 increased and less BAF60c co-precipitated compared with non-injected somites. The amount of BAF60b protein detected after CoIP was similar in control and antagomir-133-injected somites (Fig. 4H, compare lanes 1, 2). Input lanes showed a relative increase in BAF60a protein after antagomir-133 injection (Fig. 4H, compare lanes 7, 8). Inhibition of both miR-1 and miR-206 with antagomirs led to an increase in the amount of BAF60b protein that interacted with Brg1, when compared with the non-injected somites. BAF60c was detected at reduced levels in the complex after antagomir-1/206 injection (Fig. 4H, compare lanes 3, 4). Input showed a relative increase in BAF60b after antagomir-1 and -206 injection (Fig. 4H, compare lanes 9, 10). Injection of scrambled antagomir did not affect the levels of BAF60 variants that co-immunoprecipitated with Brg1, when compared with non-injected somites (Fig. 4H, compare lanes 5, 6). Input samples for scrambled antagomir are shown (Fig. 4H, compare lanes 11, 12). A similar CoIP experiment, including a negative control, is shown in supplementary material Fig. S4E. These data suggest that miR-133, miR-1 and miR-206 affect the composition of BAF/Brg1 chromatin remodeling complexes through post-transcriptional regulation of BAF60a and BAF60b variants during somite differentiation.

### Antagomir-induced inhibition of myogenesis can be restored by MO knockdown of BAF60a or BAF60b

**Fig. 5.**
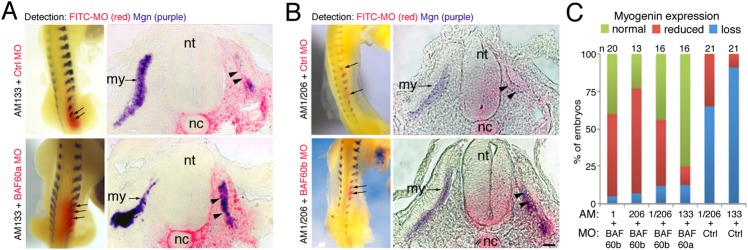
**Knockdown of BAF60a or BAF60b restores myogenesis after miRNA inhibition.** (A) Somites injected with AM133 were electroporated with FITC-labeled control-MO or BAF60a-MO, as indicated. *In situ* hybridization for myogenin transcripts (purple) and detection of FITC-MO (red) shows that myogenic differentiation is inhibited in the presence of AM133 plus control-MO (top row, arrows and arrowheads); whole-mount and section are shown. Co-electroporation of BAF60a-MO with AM133 rescued the expression of myogenin (bottom row). (B) Somites injected with AM1 plus AM206 were electroporated with FITC-labeled control-MO or BAF60b-MO, as indicated. *In situ* hybridization for myogenin transcripts (purple) and detection of FITC-MO (red) shows that myogenic differentiation is inhibited in the presence of AM1 and AM206 plus control-MO (top row, arrows and arrowheads). Myogenin expression is rescued when BAF60b-MO is co-electroporated with AM1 and AM206 (bottom row, arrows and arrowheads). Scale bar: 50 µm. (C) Summary of phenotypes observed after AM/MO injections. my, myotome; nt, neural tube; nc, notochord.

Next we examined whether BAF60a or BAF60b KD using MOs could rescue the antagomir-induced loss of myogenin in developing somites. Somites of HH14-15 embryos were injected with antagomir-133 or with antagomir-1 plus antagomir-206 and co-injected and electroporated with either control-MO or with specific BAF60a-MO (after antagomir-133 injection) or BAF60b-MO (after antagomir-1/206 injection) and analyzed after 24 h. All MOs were FITC labeled and electroporation led to a mosaic distribution. To determine the effects on differentiation, myogenin expression was examined by *in situ* hybridization. As before, embryo myogenesis was inhibited after antagomir-mediated inhibition of myomiRs in the presence of control-MO (Fig. 5A,B top panels, 5C). However, expression of myogenin was partly restored in the presence of antagomir when BAF60a or BAF60b was knocked down using specific MOs (Fig. 5A,B bottom panels, 5C).

**Fig. 6.**
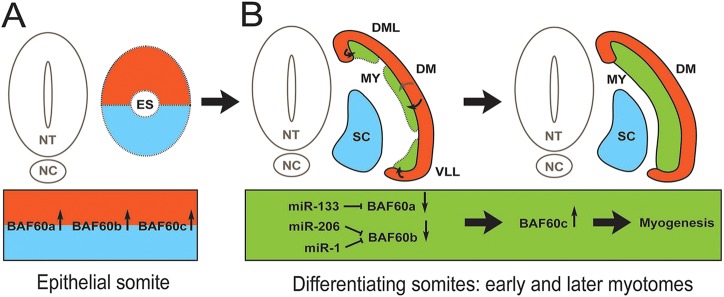
**Model illustrating the expression and regulation of BAF60 variants in embryonic somites by myomiRs.** (A) All BAF60 variants are expressed throughout epithelial somites. The coloring indicates different cell lineages; the ventral half contains chondrogenic progenitors (blue) and the dorsal half contains myogenic progenitors (red). (B) In differentiating somites miR-133, miR-1 and miR-206 are expressed in the myotome (green), which is generated from the edges of the dermomyotome (red), as indicated by arrows. We propose that myomiRs decrease the levels of BAF60a and BAF60b protein available to bind to Brg1, thus allowing an increase in BAF60c to be incorporated into the BAF/Brg1 complex. This switch in complex composition permits activation of myogenic differentiation in embryonic myocytes. The continued presence of high levels of BAF60a and BAF60b variants interferes with myogenic differentiation, presumably by displacing BAF60c from the Brg1 chromatin remodeling complex. ES, epithelial somites; DML, dorsomedial lip; VLL, ventrolateral lip; DM, dermomyotome; MY, myotome; NT, neural tube; NC, notochord; SC, sclerotome.

These results indicate that, following myotome formation and myoblast commitment, miR-133 and miR-1/206 negatively regulate BAF60a and BAF60b variants by preventing the translation of residual transcripts expressed in progenitors at earlier developmental stages. We propose that this then allows the commencement of the myogenic differentiation program in the somite myotome by affecting the composition of Brg1 chromatin remodeling factors and, in particular, the incorporation of BAF60 variants (Fig. 6).

## DISCUSSION

Mammalian BAF chromatin remodeling complexes are involved in cell differentiation and reprogramming. Together with transcription factors, BAF complexes govern cell lineage decisions, and subunit composition is thought to determine specificity. However, it is not clear how complex assembly is controlled during tissue development and embryogenesis (Wu et al., 2009; Puri and Mercola, 2012). Here, we reveal the expression of BAF60 variants in embryonic somites and uncover the negative regulation of BAF60a and BAF60b by the myomiRs miR-1/206 and miR-133 during the commitment and differentiation of embryonic myoblasts. We identify the chromatin remodeling factor BAF60a as an important target for miR-133 and show that BAF60b is an important target for miR-1/206, not only in myogenic C2C12 cells (Goljanek-Whysall et al., 2012b) but also in embryonic myoblasts in developing somites. Interference with myomiR function following myoblast commitment led to changes in BAF60a and BAF60b expression levels in somites, a concomitant switch in BAF subunit composition *in vivo* and delayed myogenic differentiation. Thus, the concerted negative regulation of BAF60a and BAF60b levels by the myomiR families is crucial to enable BAF60c-driven myogenic differentiation in the maturing myotome.

An important role has been identified for ATP-dependent chromatin remodeling during the initiation of muscle differentiation, and dominant-negative chromatin remodeling enzymes block MyoD-mediated myogenic differentiation of NIH3T3 fibroblasts (de la Serna et al., 2001). These effects correlated with changes in myogenin promoter chromatin structure and with altered expression levels of a number of other muscle differentiation genes (de la Serna et al., 2005). Thus, it will be interesting to determine how different BAF60 variants affect the activity of the Brg1 complex, its binding to muscle-specific transcription factors and effects on chromatin structure at native muscle promoters.

BAF60c is important for cardiac and skeletal myogenesis (Lickert et al., 2004; Ochi et al., 2008; Forcales et al., 2012) and has been shown to play a role during smooth muscle differentiation (Sohni et al., 2012). miRNA-mediated regulation provides a mechanism by which the composition of the BAF/Brg1 complex can be controlled at the post-transcriptional level during skeletal muscle development. Interestingly, miRNA-mediated exchange of BAF variants was described during neuronal differentiation, where BAF53a is downregulated by miR-9* and miR-124 (Yoo et al., 2009). This suggests that miRNA-regulated switching of BAF subunit composition might be a common regulatory mechanism in both myogenic and neurogenic lineages. A similar circuitry has recently been revealed in fibro-adipogenic cells (FAPs) in postnatal muscle. Treatment with HDAC inhibitors led to upregulation of MyoD, BAF60c and myomiRs, which affected the levels of BAF60a and BAF60b variants and altered FAP myogenic potential (Saccone et al., 2014).

Exploiting the accessibility of the chicken embryo for *in vivo* manipulations, we previously identified a crucial role for miR-206 during the transition of myogenic progenitors to committed myoblasts. We showed that negative regulation of the pro-myogenic, paired-box transcription factor Pax3 ensures robust execution of this developmental program in early somites (HH12) (Goljanek-Whysall et al., 2011). The present study extends this work to assess the role of the miR-1/206 and miR-133 cluster in later somite differentiation (HH14-15). We propose that these muscle-specific miRNAs, which are activated by MRFs (Rao et al., 2006; Rosenberg et al., 2006; Sweetman et al., 2008), serve to stabilize the differentiation program of committed myoblasts (Fig. 6). This is achieved through targeting of crucial genes, including *Pax3* and *BAF60a*/*b*, which are associated with progenitor states of muscle cells or with potential alternative differentiation paths.

Consistent with the latter, previous work showed that sustained expression of BAF60b in C2C12 myogenic cells inhibited skeletal muscle differentiation and enabled the expression of genes associated with alternative, non-myogenic lineages, including chondrogenesis and osteogenesis (Goljanek-Whysall et al., 2012b). Interestingly, BAF60a, but not BAF60c, has been shown to be present in ESC BAF (esBAF) complexes, which are required for self-renewal and pluripotency of mouse ESCs (Ho et al., 2009). Moreover, BAF60a was shown to be a part of a BAF complex in cardiac progenitors and suggested to be exchanged for BAF60c during cardiac differentiation (Chen et al., 2012). Here we demonstrate that targeted misexpression of BAF60a or BAF60b variants or antagomir-mediated inhibition of miRNA function in somites inhibited myogenic differentiation of committed myoblasts. Antagomir injection led to a specific increase of either BAF60a or BAF60b, in terms of both protein and transcripts (Fig. 4D-G; supplementary material Fig. S4F), and this affected the BAF/Brg1 complex composition, potentially by displacement of BAF60c from Brg1. However, additional cross-regulation of BAF60a, BAF60b and BAF60c expression cannot be excluded at present.

miRNAs and their targets are often expressed in a mutually exclusive fashion (Hornstein and Shomron, 2006; Ebert and Sharp, 2012). During somite development, BAF60a, BAF60b and BAF60c are expressed in the epithelial somite. The epithelial somite undergoes dramatic reorganization and differentiates into the dermomyotome and myotome, with the dermomyotome layer containing the progenitors, which will translocate into the myotome (Gros et al., 2004). During somite differentiation, all BAF60 variants were detected in the myotome (Fig. 1B) and qPCR revealed an increase in *BAF60c* in older somites compared with *BAF60a* and *BAF60b* (Fig. 1C). CoIPs showed an increased amount of BAF60c bound to Brg1 compared with BAF60a and BAF60b (Fig. 1D), suggesting a shift in BAF/Brg1 complex composition during somite differentiation. Our data indicate that myomiR inhibition interferes with this process (Fig. 4H; supplementary material Fig. S4E), possibly through derepression of *BAF60a* and *BAF60b* transcripts present in the myotome.

It has been shown that BAF60c directly interacts with MyoD in both undifferentiated and differentiating C2C12 myoblasts. In response to p38 signals, the BAF complex is recruited to activate the transcription of muscle genes (Forcales et al., 2012). We show here for the first time that the amount of BAF60a and BAF60b variants bound to Brg1 decreases during somite differentiation, and our experiments suggest that negative post-transcriptional regulation, mediated by miR-1/206 and miR-133, is necessary for the timely progression of myogenic differentiation. The data support the idea of a BAF60 variant switch during embryonic myogenesis. We show that BAF60a and BAF60b are dispensable for myogenic differentiation *in vivo* (supplementary material Fig. S2A,B), and their elevated expression in developing somites led to changes in BAF/Brg1 complex composition and adversely affected differentiation (Fig. 2D-F, Fig. 4H; supplementary material Fig. S4E). MO-mediated rescue experiments suggest that, at this stage in development, delayed myogenin expression induced by myomiR inhibition is largely related to elevated levels of BAF60a and BAF60b (Fig. 5). It remains to be established whether this correlates with structural chromatin changes at the myogenin promoter (de la Serna et al., 2001, 2005).

Chicken BAF60a and BAF60b 3′UTR sensor constructs containing target sites for either miR-133 or miR-1/206 were efficiently targeted by miR-133 or miR-1/206 (Fig. 3C; supplementary material Fig. S3C). In mouse NIH3T3 cells, endogenous BAF60a and BAF60b expression was regulated by the myomiRs at the level of protein and RNA (Fig. 3D; supplementary material Fig. S3B), suggesting effects on both mRNA stability and repression of protein translation. Interestingly, the miR-1 and miR-206 seed sequences are not well conserved in the 3′UTR of human and mouse *BAF60b* (Fig. 3A). This indicates species-specific differences and suggests the presence of non-canonical miR-1/206 target sites in mouse and human. Non-canonical miRNA binding sites use additional complementary nucleotides outside the seed sequence, are widespread and are able to regulate gene expression (Loeb et al., 2012).

BAF60c, together with the cardiac transcription factors Gata4 and Tbx5 can direct the ectopic differentiation of mouse mesoderm into beating cardiomyocytes. Interestingly, BAF60b was able to replace BAF60c in this assay, although BAF60b was less efficient at driving reprogramming (Takeuchi and Bruneau, 2009). In skeletal muscle, BAF60b has been proposed to have an ‘ancillary' function to BAF60c (Puri and Mercola, 2012) and both variants have been identified as MyoD-interacting proteins in yeast two-hybrid assays (Forcales et al., 2012), suggesting a degree of redundancy. Our data support the role of BAF60c as the ‘master’ BAF60 variant necessary for muscle differentiation. It remains possible that BAF60a and BAF60b variants are important in progenitors. Their expression in somites is consistent with this, and it will be interesting to determine which factors associate with BAF60a- and BAF60b-containing complexes in progenitors and early myoblasts. It remains to be seen which genes are regulated by these complexes during lineage specification; but it is clear that negative regulation of BAF60a and BAF60b variants is important. The loss of negative regulation led to impaired myogenesis in embryos.

Our results are consistent with the proposed function of miRNAs in the fine-tuning of genetic programs (Hornstein and Shomron, 2006; Ebert and Sharp, 2012) and suggest that the coordinated downregulation of BAF60a and BAF60b variants by myomiRs provides robustness to the muscle differentiation program through effects on Brg1/BAF60 variant composition. It appears that this is not only important in embryo myogenesis, but also postnatally, when it can affect the myogenic potential of FAPs (Saccone et al., 2014).

## MATERIALS AND METHODS

### DNA constructs, transfections and luciferase assay

Sensor constructs contained chick *BAF60a* or *BAF60b* 3′UTR fragments in a modified pGL3 vector (Promega); for primers see supplementary material Table S1 and Goljanek-Whysall et al. (2011). Mutant constructs had *Bam*HI or *Sal*I sites within miR-1/206 or miR-133 target sites. Chick dermal fibroblasts (DF1) were transfected with 200 ng plasmid with or without miRNAs (50 nM) (Sigma) using Lipofectamine 2000 (Invitrogen) in 96-well plates. miRNA mimics were identical to endogenous miRNAs; for sequences see supplementary material Table S1. pGL3 vector without 3′UTR or with mutant 3′UTRs, or the transfection of unrelated miR-140, served as a negative control. Transfections employed triplicate samples and were repeated four times using independent plasmid preparations. Firefly and Renilla luciferase activities were measured after 48 h using a multi-label counter (Victor2, PerkinElmer) and relative activities were calculated. Mouse BAF60a and BAF60b expression vectors (MRC Geneservice) and GFP plasmid were used for targeted misexpression *in vivo* at a ratio of 5:1.

### Cell culture, western blot and qPCR

Mouse NIH3T3 cells in DMEM, 10% FBS, 1% pen/strep were transfected with miR-206, miR-1 or miR-133 (50 nM; Sigma) with and without antimiRs (100 nM; Ambion) using Lipofectamine 2000. Mock-transfected cells served as controls. Protein was extracted after 48 h and 20 μg was run on 8-12% polyacrylamide gels and blotted onto PVDF membrane. Primary antibodies (Abcam) to BAF60a (1:500; ab83208), BAF60b (1:1000; ab81622), BAF60c (1:500; ab50556), Brg1 (1:500; ab110641) and actin (1:1000; ab3280) were applied at 4°C overnight; secondary antibodies (Dako, P0447; and Jackson ImmunoResearch, 111-035-003) were applied for 1 h at room temperature.

RNA was isolated with TRIzol (Invitrogen). cDNA synthesis used SuperScript II reverse transcriptase (Invitrogen) with 1 μg RNA (cells) or 400 ng RNA (somites). For miRNA qPCR, the MystiCq miRNA cDNA Synthesis Kit (Sigma) was used. Primers (supplementary material Table S1), miRNA primers (designed by Sigma) and SYBR Green MasterMix (Applied Biosystems, Sigma) were used with the Applied Biosystems 7500 Fast Real-Time PCR System following the manufacturer's protocols. All qPCR was normalized to beta-actin (mRNA) or *RNU-6* (miRNA).

### *In situ* hybridization and immunohistochemistry

Whole-mount *in situ* hybridization using double DIG-labeled LNA oligos (Exiqon) or antisense RNA probes was carried out as previously described (Goljanek-Whysall et al., 2011). Probes detecting BAF60 variants were designed to overlap 3′UTR regions to ensure specificity. Embryos were fixed in 4% paraformaldehyde, embedded in O.C.T. Compound (Sakura Finetek), sectioned and immunostained as described (Goljanek-Whysall et al., 2011). Primary antibodies (Abcam; see above) were used at 1:100 dilution and incubated at 4°C overnight; secondary antibody (Molecular Probes, A11008) was used at 1:500 dilution. DAPI (Sigma) was used to stain cell nuclei (1:10,000 dilution).

### Injection of antagomir and MOs

Antagomirs (Dharmacon) were designed based on published methods (Goljanek-Whysall et al., 2011). All bases were 2′O-methyl bases; thiol bonds replaced phosphodiester bonds between bases 1-2, 2-3, 19-20, 20-21 and 21-22; 3′ cholesterol was added. Scrambled sequences were used as controls [final concentration 1 mM, except when mixed with MOs (1:1) in rescue experiments]. The posterior six somites of HH14-15 embryos were injected. Embryos were harvested after 24 h and injected somites and corresponding somites from the uninjected side were dissected and lysed. Somites from 20-25 embryos were pooled for western blot analysis; three biological repeats used material from independent experiments.

BAF60 MOs were 3′ FITC labeled (Gene Tools) (supplementary material Table S1). Control MOs do not target any known gene. MOs were injected into somites at HH14-15 and embryos were electroporated using six 10 msec pulses of 60 V. Embryos were harvested after 24 h for analyses. Mouse BAF60a or BAF60b expression vectors mixed with a GFP plasmid in a 5:1 ratio or GFP expression vector alone were electroporated as described for MOs.

### Co-immunoprecipitation

Somites of non-treated HH12 or HH20 embryos were dissected and lysed. Somites from embryos injected at HH14-15 with antagomirs were dissected after 24 h, pooled and lysed in 20 mM HEPES (pH 7.4), 150 mM NaCl, 10% glycerol, 1% Triton X-100 on ice for 15 min. We obtained 20 μg protein from untreated somites and 10 μg from antagomir-injected somites. The supernatant was split for immunoprecipitation (40%), input (20%) and negative control (40%) samples. Supernatants were precleared with pre-blocked protein A-agarose (Sigma, P1406) on ice, with agitation for 1 h. Binding reactions were performed with 10 μl anti-Brg1 antibody (see above) or rabbit IgG (Abcam, ab27478) on ice with agitation for 2 h, and for an additional 2 h with 15 μl pre-blocked protein A-agarose. Bound immune complexes were washed three times in 20 mM HEPES (pH 7.4), 150 mM NaCl, 10% glycerol, 0.1% Triton X-100 and resuspended in 10 μl 1× Laemmli buffer, boiled and run on 8-12% polyacrylamide gels (Bio-Rad), followed by western blot.

## Supplementary Material

Supplementary Material
