# Supplementary material for: myomiR-dependent switching of BAF60 variant incorporation into Brg1 chromatin remodeling complexes during embryo myogenesis
Source: Development. 2014 Sep;141(17):3378–87. doi: 10.1242/dev.108787 (PMC4199139; doi:10.1242/dev.108787)
Supplement: Supplementary Material [file supp_141_17_3378__index.html]

myomiR-dependent switching of BAF60 variant incorporation into Brg1 chromatin remodeling complexes during embryo myogenesis — Supplementary Material 

# myomiR-dependent switching of BAF60 variant incorporation into Brg1 chromatin remodeling complexes during embryo myogenesis

## DEV108787 Supplementary Material

**Files in this Data Supplement:**

- **Supplementary Material**
