# Supplementary material for: myomiR-dependent switching of BAF60 variant incorporation into Brg1 chromatin remodeling complexes during embryo myogenesis
Source: Development. 2014 Sep;141(17):3378–87. doi: 10.1242/dev.108787 (PMC4199139; doi:10.1242/dev.108787)
Supplement: Supplementary Material [file supp_141.17.3378_DEV108787.pdf]

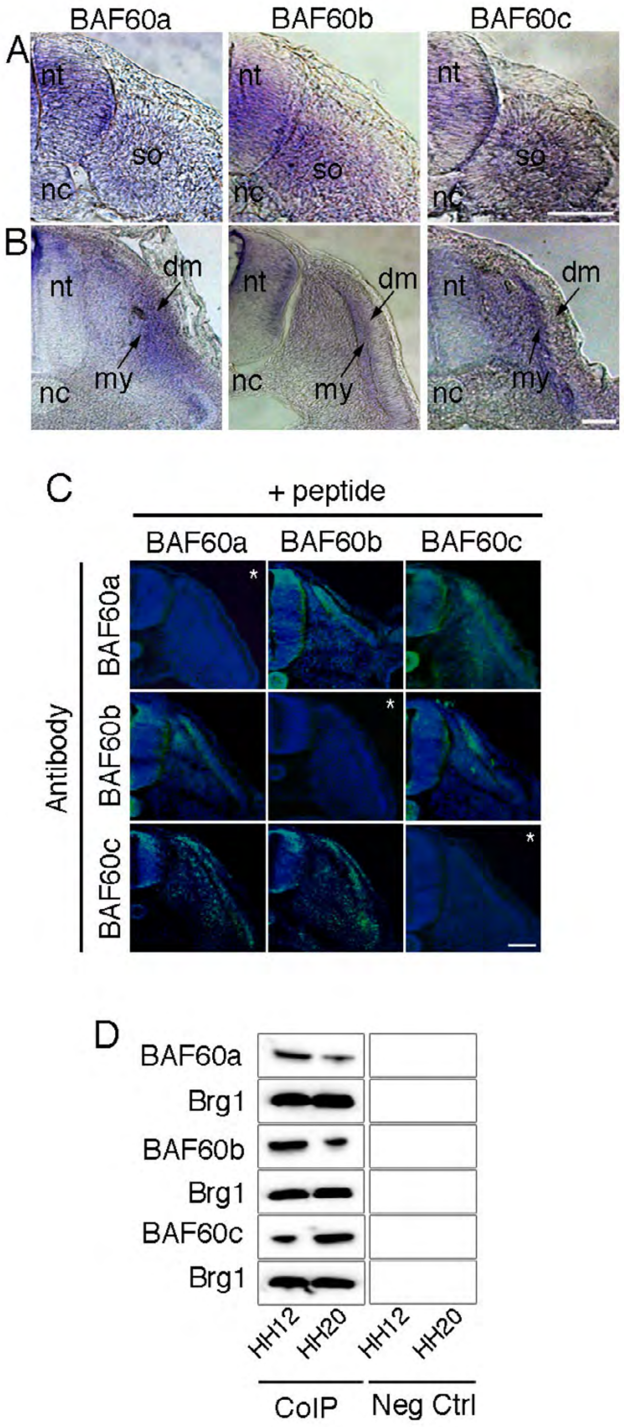

Fig. S1. Expression of BAF60 variants in developing somites. *In situ* hybridization using specific antisense RNA probes against the divergent 3'UTR of BAF60a, -b, and –c is shown. (A) Transcripts encoding all three variants are detected in epithelial somites of HH12 embryos. (B) In differentiating somites of HH20 embryos, BAF60a, -b and -c transcripts are detected in the myotome (my). (C) For peptide blocking, primary antibodies (Abcam; see Materials and Methods) were used at 1:500 dilution and mixed with 1 µg/ml peptide (Covalab; see supplemental table 1). Antibody/peptide mixtures were incubated overnight at 4°C. Immunohistochemistry was then performed as stated in materials and methods. When BAF60a antibody was incubated with BAF60a peptide, or BAF60b antibody was incubated with BAF60b peptide, or BAF60c antibody was incubated with BAF60c peptide (indicated by *) there was a complete loss of signal in the neural tube and myotome. When BAF60a, -b and –c antibodies were incubated with other peptides, signal was present in the neural tube and myotome. All images were exposed for the same mount of time. Cell nuclei stained with DAPI (blue) and the BAF60 variants stained in green. Scale bars 50 µm. (D) CoIP using Brg1 antibody and protein isolated from HH12 or HH20 somites shows that the amount of BAF60a and BAF60b protein bound to Brg1 decreases over time, whereas the amount of BAF60c variant associated with Brg1 increases in differentiating somites. IgG antibody-only samples were used as negative control. nt, neural tube; nc, notochord; so, somite; dm, dermomyotome.

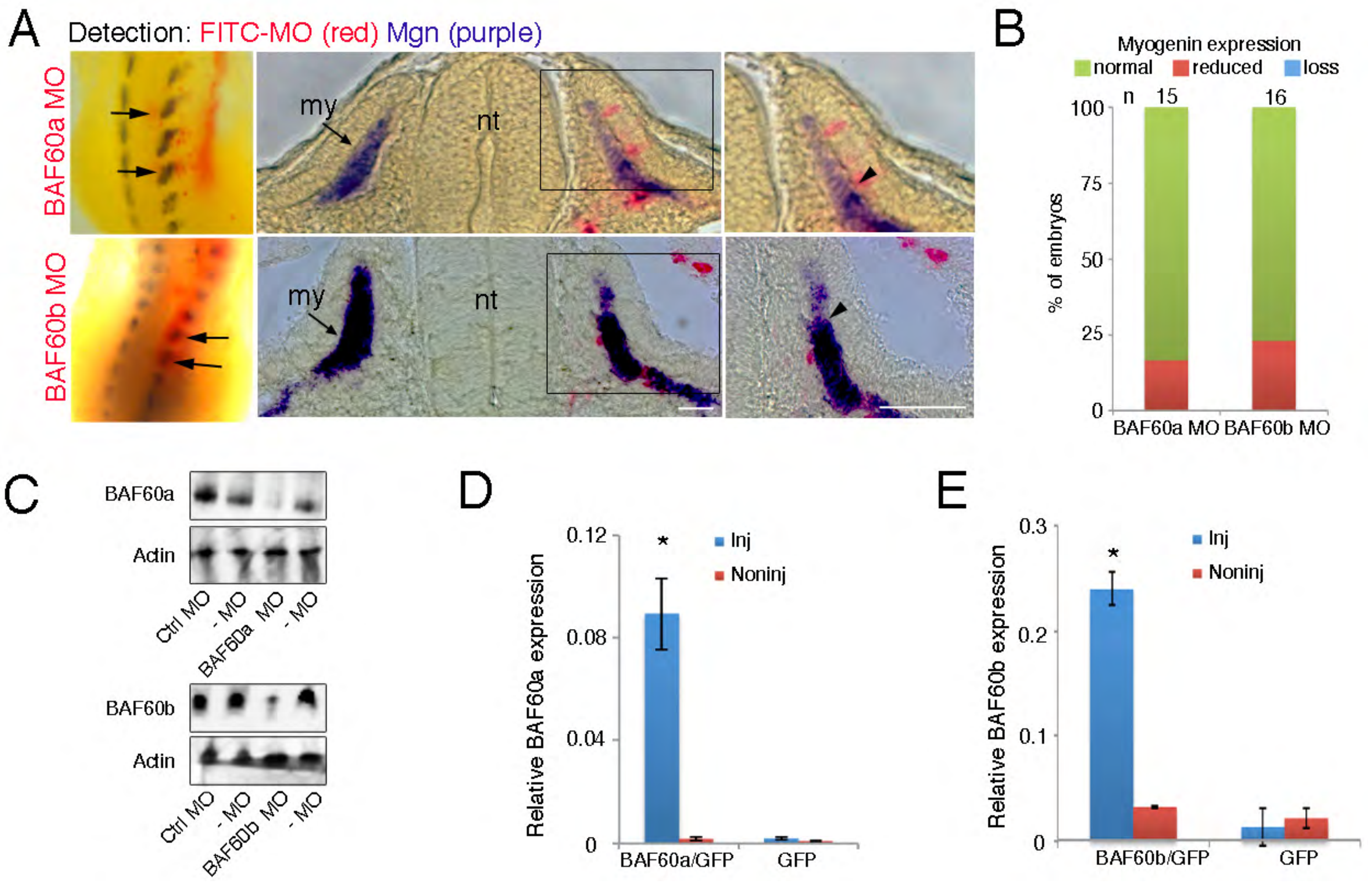

Fig. S2. Knock-down of BAF60a or BAF60b does not affect myogenin expression. (A) Double whole mount *in situ* hybridization for myogenin (purple, arrows and arrowheads) and detection of FITC-labeled morpholinos (red) against BAF60a or BAF60b, as indicated. Sections show myogenin expression on the MO-injected side is largely unaffected compared to the contralateral side. (B) Summary of myogenin phenotypes observed after MO injections: green, normal; red, reduced. In some embryos reduced expression was observed. (C) Western blot of protein extracted from somites injected with control-MO or BAF-MO as indicated, -MO indicates non-injected, contralateral somites. Reduced levels of BAF60a protein were seen after BAF60a-MO injection; reduced levels of BAF60b protein were observed after BAF60b-MO injection. Electroporation of MOs is mosaic and not all cells are affected, the WB indicates the average across the pooled tissue. (D) qPCR detecting increased amounts of BAF60a transcripts in somites electroporated with BAF60a expression construct along with a GFP tracer (ratio of 5:1) compared to contralateral non-injected somites. No increase was seen in GFP only electroporated somites. Error bars are SD (*P<0.05, T-test). (E) qPCR detecting increased amounts of BAF60b transcripts in somites electroporated with BAF60b expression construct along with a GFP tracer (ratio of 5:1) compared to contralateral, non-injected somites. Injection and electroporation of GFP only served as control and no effect on BAF60b transcripts was observed Error bars are SD (*P<0.05, T-test).

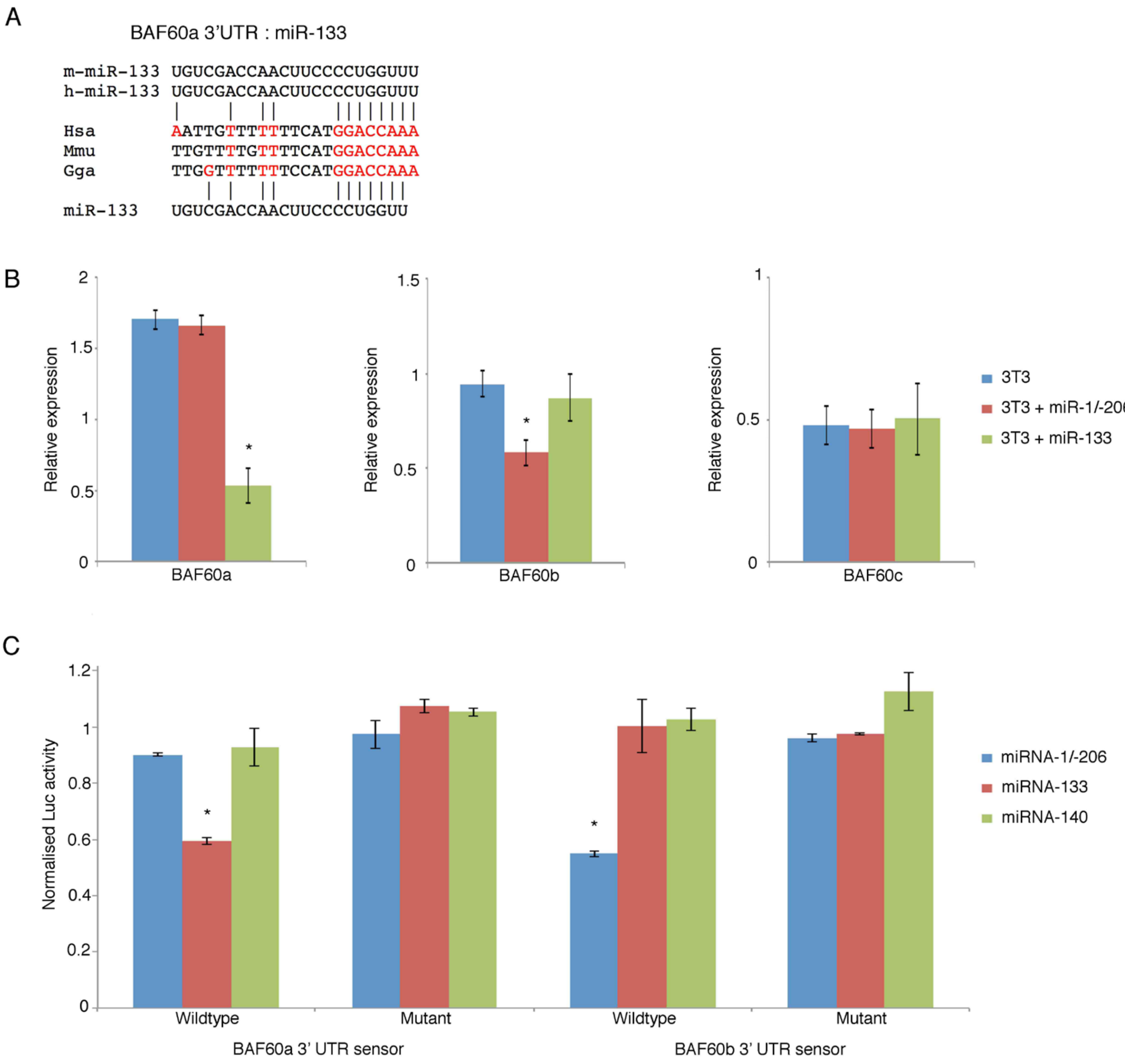

Fig. S3. myomiRs regulate expression of BAF60 variants in mouse NIH3T3 cells. (A) Alignment of the putative miR-133 target site in the 3'UTRs of chick (*Gallus gallus*, Gga), human (*Homo sapiens*, Hsa) and mouse (*Mus musculus*, Mmu) BAF60a gene. Nucleotides complementary to the respective miR are colored red. The human (h-mir-133) and mouse (m-mir-133) sequences contain an additional uracil at the 5' end, which is complimentary to the target sequence. (B) qPCR of mouse NIH3T3 cells for BAF60a, BAF60b, or BAF60c. Cells were mock transfected (blue) or transfected with miR-1 and miR-206 (red) or with miR-133 (green). The expression of endogenous BAF60a was affected by miR-133, but not by miR-1 and miR-206. The expression of endogenous BAF60b was affected by miR-1 and miR-206, but not by miR-133. The expression of endogenous BAF60c transcripts was not affected by transfection of myomiRs. Error bars are SD (*P<0.05, T-test). (C) Luciferase sensors containing 3'UTR sequences of chick BAF60a or BAF60b were transfected into DF-1 cells. Transfection of miR-133 led to a significant down-regulation of luciferase expression from the BAF60a 3'UTR sensor compared to controls, however transfection of miR-1 and miR-206 had no effect. Transfection of a non-related miRNA, miR-140, served as a negative control. Point mutations in the putative target site rendered the sensor non-responsive to miR-133 Error bars are SD (*P<0.05, T-test). Co-transfection of miR-1 and miR-206 led to significant down-regulation of luciferase expression from the BAF60b 3'UTR sensor compared to controls, however transfection of miR-133 had no effect. Transfection of a non-related miRNA, miR-140, served as a negative control. Point mutations in the putative target site rendered the sensor non-responsive to miR-1 and miR-206.

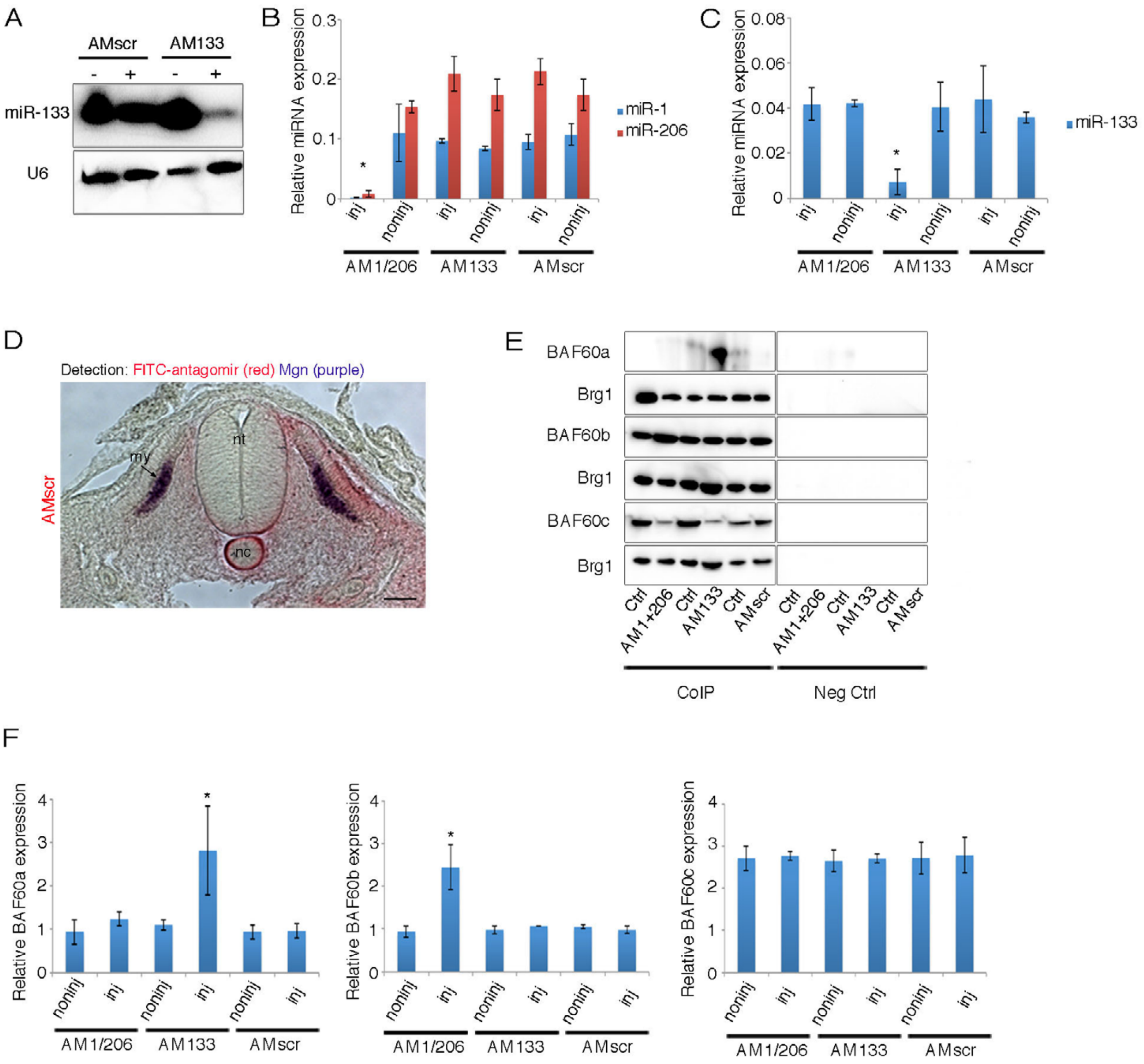

Fig. S4. Antagomir-133 and antagomir-1/206 downregulate miR-133 and miR-1/206 respectively and results in upregulation of BAF60a and BAF60b transcript and protein. (A) Northern blot of somites injected with scrambled antagomir (AMscr) or with antagomir-133 (AM133), both indicated with (+), shows specific effects on miR-133. Contralateral non-injected somites are shown, indicated by (-). (B) qPCR detecting miR-1 (blue) or miR-206 (red) in somites injected with antagomirs as indicated. AM1 and AM206 resulted in almost complete loss of miR-1 and miR-206, but expression of these miRNAs was not affected by AM133 or AMscr. (C) qPCR detecting miR-133 in somites injected with antagomirs as indicated. AM133 significantly reduced miR-133 expression, however AM1/206 or AMscr had no effect. Error bars are SD (*P<0.05, T-test). (D) Section of somites injected with AMscr shows that there is no effect on the expression of myogenin after 24 hours. Myogenin (purple) is detected by *in situ* hybridization using antisense RNA probe. The FITC-labeled AM is detected using an anti-FITC antibody coupled to alkaline phosphatase (red). Scale bar 50 mm. my, myotome; nt, neural tube; nc, notochord. (E) CoIP using anti-Brg1 antibody or IgG only as negative control. Somites were injected with relevant antagomirs, AM1 and AM206, or AM133, as indicated. After 24 hours somites were dissected together with non-injected somites from the opposite side (Ctrl) for protein lysis. Western blot detected similar Brg1 amounts in all lanes. More BAF60a protein and less BAF60c protein were pulled down with Brg1 after AM133 injection; more BAF60b protein and less BAF60c protein was pulled down with Brg1 after injection with AM1 and AM206. This suggests that myomiR inhibition affects BAF/Brg1 complex composition. (F) qPCR for transcripts of BAF variants expressed in somites after injection with relevant antagomirs. AM133 led to an increase of BAF60a transcripts, but AM1/206 or AMscr did not. AM1/206 led to an increase of BAF60b transcripts, but AM133 or AMscr did not. BAF60c transcript levels in embryonic somites were not affected by any of the antagomirs injected. Error bars are SD (*P<0.05, T-test).

Table S1: microRNA mimics, primers, morpholinos and peptides

miR-1	5′-UGGAAUGUAAAGAAGUAUGUA-3′<br>5′-CAUACUUCUUUAUAUGCCCAUA-3′
miR-206	5′-UGGAAUGUAAGGAAGUGUGUGG-3′<br>5′-ACAUGCUUCUUUAUA UCCCCAUA-3′
miR-133	5'-UUGGUCCCCUUCAACCAGCUGU-3'<br>5'-AGCUGGUAAAAUGGAACCAAA U-3'
miR-140	5' AGUGGUUUUACCCUAUGGUAG 3'<br>5' CCACAGGGUAGAACCACGGAC 3'
chBAF60a RT F	GCGGCCTATCCGAGACCAGG
chBAF60a RT R	GGCCAGACCAGGTCGGACTGA
chBAF60b RT F	CCAAGGTCCAGCAGCGTCGG
chBAF60b RT R	TGGGGCGGGGAGAATCAGGG
chBAF60c RT F	AGTCCCAGGCCTACATGGAT
chBAF60c RT R	TCCGCTTTTGCTTCATTGGC
chBeta-actin RT F	CCAGCTGGGAGGAGCCGGT
chBeta-actin RT R	CTGGGGAACACAGCCCGCTT
chMyogenin RT F	GCCATCCAGTACATCGAGCG
chMyogenin RT R	CTCAGGAGGTGATCTGCGG
mBAF60a RT F	GGTCCAAAATCGAAATCACAATGC
mBAF60a RT R	GGACCAGTTCCCGAATCCTT
mBAF60b RT F	CTTCTGGAGGCATGGGGGTA
mBAF60b RT R	ATGCCAGGACGCTGGTACT
mBAF60c RT F	AACGCAGGGCTGAGTTCTAC
mBAF60c RT R	CTGCGCTGCTGGATCTTACA
mBeta-actin RT F	GATCAAGATCATTGCTCCTCCTG
mBeta-actin RT R	AGGGTGTAAAACGCAGCTCA
chBAF60a 3'UTR F	AGATCTGCCCTCATCTCCTCCCCACATT
chBAF60a 3'UTR R	GCTAGCAGGAGGACAGCTTCCTTCACAG
chBAF60b 3'UTR F	AGATCTGCCTGACGGTACTTCTTACTG
chBAF60b 3'UTR R	GCTAGCGCTGGCATTTGGAATGACAAAC
chBAF60a probe F	GCCCTCATCTCCTCCCCACATT
chBAF60a probe R	AGGAGGACAGCTTCCTTCACAG
chBAF60b probe F	CGTACCCCTGGGCTCCCCTC
chBAF60b probe R	GCCAGGAGGGCTTTGGCCAG
chBAF60c probe F	CCTAGCCTGGCACAGGGCTAT
chBAF60c probe R	TCACAGTCTGGGGCATCCCAGG
BAF60a Morpholino (translation blocking)	GCCGCCATCTTCTCCAGCACAAAGA
BAF60b Morpholino (e2i2 splice junction target)	GCTCCCGTGTCACCCCTTACCCTCT
BAF60c Morpholino (splice junction targets)	TGTGGCTAACTCCTTCATACCTTGC (e13i13)
Control Morpholino	CCTCTTACCTCAGTTACAATTTATA
cBAF60a peptide	RKLRIFISNTFNPAKSDAEDGEGTVASWELRVEGRLLEDSALSK YDATKQ
cBAF60b peptide	SNNPQDFIQEWIKSQRRDLKIITDVIGNPEEERRAEFYQQPWA QEAVGRH
cBAF60c peptide	FERKLDQTIMRKRVDIQEALKRPMKQKRKLRLYISNTFNPAKSD ADDSDG
